# First Example of Cage P_4_N_4_-Macrocycle Copper Complexes with Intracavity Location of Unusual Cu_2_I Fragments

**DOI:** 10.3390/molecules28020680

**Published:** 2023-01-09

**Authors:** Anna S. Balueva, Yulia A. Nikolaeva, Elvira I. Musina, Igor A. Litvinov, Andrey A. Karasik

**Affiliations:** Arbuzov Institute of Organic and Physical Chemistry, FRC Kazan Scientific Center, Russian Academy of Sciences, Arbuzov Str. 8, 420088 Kazan, Russia

**Keywords:** cyclophanes, cyclic aminomethylphosphines, copper complexes, intracavity, crystal structure

## Abstract

In this study, 28-membered macrocyclic 1,5(1,5)-di(1,5-diaza-3,7-diphosphacyclooctana)-2,4,6,8(1,4)-tetrabenzenacyclooctaphane were synthesized by condensation of pyridinephosphine, paraformaldehyde, and primary diamines (bis(4-aminophenyl)methane or -sulfide. The first representatives of binuclear copper(I) complexes of P,N-containing cyclophanes with two 1,5-diaza-3,7-diphosphacyclooctane rings incorporated into a macrocyclic core and intracavity location of unusual, developed angle Cu_2_I moiety were obtained. The structure of one complex was established by X-ray diffraction analysis. The complexation led to a slight distortion of the cyclophane conformations.

## 1. Introduction

A distinctive feature of phosphorus-containing cyclophanes is the combination of two binding sites with different features. The first site is a phosphorus atom that is able to form rather stable coordination bonds with various soft transition metal ions in a low oxidation state. The second site is a hydrophobic intramolecular cavity bounded by phenylene fragments which is suitable for host–guest interactions with small organic molecules or for encapsulation of a metal-containing fragment connected with a phosphorus atom. This explains why phosphorus-containing cyclophanes could be regarded to be a promising scaffold for tailor-made transition metal complexes which may be of interest both for homogeneous catalysis and for the creation of novel recognition systems and molecular materials [1,2,3,4,5,6,7,8,9,10].

However, there are only a few examples of intracavity location of transition metals connecting with phosphorus atoms [1,2,11,12]. Gyroscope-like compounds with platinum(II) containing rotators encased in macrocyclic diphosphines have been obtained via intramolecular alkene metatheses [13,14]. It has been shown that five-coordinate rhodium(III) complexes of N,P_2_-pincer macrocyclic ligands were useful synthons for the effective synthesis of dihydrogen, ethylene, and carbonyl derivatives of rhodium(I) [15]. The high activity and excellent enantioselectivity of iron complexes of 22-membered P,N-containing cyclophane should be mentioned [16]. The unusual stability and electrochemical behavior of a copper(II) moiety encapsulated in a cavity of a calixarene-based P,N_3_-containing ligand have been demonstrated [17,18]. Therefore, the transition metal complexes of P-containing cyclophanes, in part due to their low synthetic availability, have been studied less as compared with the analogous complexes of P-containing corands [1,2,11,12].

Various 28-, 36-, 38- and 46-membered P,N-containing cyclophanes with 1,5-diaza-3,7-diphosphacyclooctane fragments incorporated into the macrocyclic backbone have been obtained by stereoselective covalent self-assembly between primary phosphines, formaldehyde, and diamines with spatially divided amino groups [19,20,21,22,23,24,25]. Later, their coordination properties towards metals of groups VI, VIII, and X have been studied [26,27,28,29]. In the uncoordinated cyclophanes, the eight-membered 1,5-diaza-3,7-diphosphacyclooctane fragments adopt a “crown” conformation in which the phosphorus electron lone pairs are directed inside the macrocycle cavity [20,22,23,24,25]. It has been shown that complex formation with groups VI and VIII metals of these macrocycles led to dramatic changes in the initial conformations of the ligands; thus, the metal-containing moieties of the forming binuclear bis-P,P-chelate complexes were located outside the macrocyclic cavities [26,27,28,29]. Recently, it has been demonstrated that P,N-containing cyclophanes with two 1,5-diaza-3,7-diphosphacyclooctane rings incorporated into the macrocyclic core form tetranuclear gold(I) complexes with gold(I) chloride with two of the four gold(I) ions located above and below the partially collapsed macrocyclic cavity [30].

An NMR investigation as well as DFT calculations of the conformational behavior of chelate transition metal complexes with 1,5-diaza-3,7-diphosphacyclooctanes have established that the initial crown conformation was predominant for copper(I) complexes [31]. Therefore, copper(I) ion is the best candidate for the synthesis of coordination complexes with an intracavity location of the central transition metal. P-pyridyl substituted 28-membered cyclophanes with rigid phane fragments were chosen as the first targets due to the satisfactory solubility of these cyclophanes in various organic solvents. We presupposed that P,N-containing cyclophanes would form complexes with copper(I)-containing fragments located inside their hydrophobic cavities.

In the present work, we describe the synthesis and structure of the first representatives of the binuclear copper(I) complexes of 28-membered P,N-containing cyclophanes with unusual Cu_2_I fragments in the intracavity location.

## 2. Results and Discussion

### 2.1. Synthesis

**Scheme 1:**
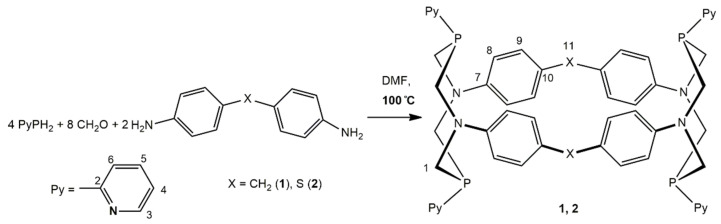
Synthesis of 28-membered cyclophanes **1** and **2**.

New macrocyclic 1,5(1,5)-di(1,5-diaza-3,7-diphosphacyclooctana)-2,4,6,8(1,4)-tetrabenzenacyclooctaphane ligands **1** and **2** were obtained by condensation reactions of bis(hydroxymehyl)(pyridine-2-yl)phosphine (prepared in situ from (pyridine-2-yl)phosphine and paraformaldehyde) and corresponding primary diamine (bis(4-aminophenyl)methane or -sulfide) in DMF, according to the method in the literature (Scheme 1) [22]. 

The yields of P-pyridine-2-yl substituted cyclophanes **1** and **2** were moderate (23 and 15%, respectively) and noticeably lower than the yields of analogous P-aryl substituted macrocycles (18–60%) [22], but close to the yields of 28-membered P-(2-(pyridine-2′-yl)ethyl) substituted macrocycles (17–21%) [32]. The elemental analysis, mass spectrometry, ^31^P and ^1^H NMR spectroscopy data of compounds **1** and **2** are consistent with their cyclophane structures which include two 1,5-diaza-3,7-diphosphacyclooctane rings. The proton signals of the P-CH_2_-N fragments of both compounds appear to be typical (AB)_2_X systems for the chair–chair conformation of the heterocyclic eight-membered fragments with the equatorial positions of substituents at the phosphorus atoms and the axial positions of their lone electron pairs, namely, two doublets of doublets with the values of the coupling constants ^2^*J*_HH_ = 15.0–15.1 Hz and ^2^*J*_PH_ = 9.7–10.5 Hz for equatorial protons and 5.6–5.7 Hz for axial protons [22,23,32]. These structures are typical for 28-membered 1,5(1,5)-di(1,5-diaza-3,7-diphosphacyclooctana)-2,4,6,8(1,4)-tetrabenzenacyclooctaphanes where the phenylene rings and 1,5-diaza-3,7-diphosphacyclooctanes form a truncated rhombohedral prism [22,32], and the conformation of diphosphine fragments is favorable for the formation of bis-P,P-chelate metal complexes.

**Scheme 2:**
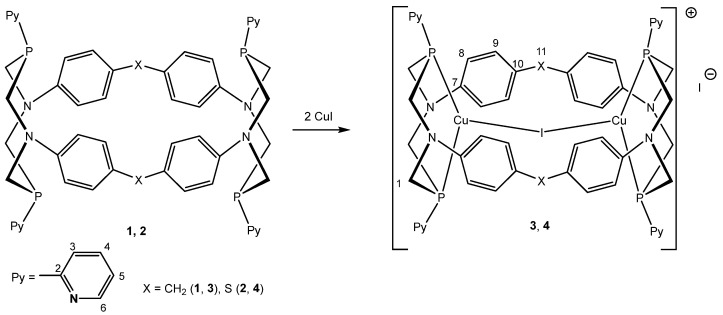
Synthesis of copper(I) complexes of 28-membered cyclophanes **3** and **4**.

The reaction of 28-membered cyclophanes **1** and **2** with two equivalents of copper(I) iodide in DMF at 100 °C results in the formation of complexes **3** and **4** (Scheme 2). 

In the spectra of the reaction mixtures, peaks are observed at δ_P_ −29.7 and −27.7 ppm for complexes **3** and **4**, respectively. A small amount of fine precipitate formed after heating the reaction mixture up to 100 °C, for two days. The very low solubility of this powder did not allow us to establish the structure of the compound. The pale yellow powders of complexes **3** and **4** were obtained using the following concentration of the filtrate, in moderate yields of 43% and 48%, respectively. Complexes **3** and **4** were soluble in DMF and DMSO. The elemental analysis data for both isolated products were consistent with their formula LCu_2_I_2_ (L = **1**, **2**). The ESI mass spectra show the most intensive peaks with *m/z* 1191 (**3**) and 1227 (**4**) for the cations of the composition [LCu_2_I]^+^ along with additional peaks [LCu_2_ + Na]^+^ (for both **3** and **4**) and [LCu_2_ + 2Na]^+^ and [LCu_2_ + O + (CH_3_)_2_SO]^+^ (for **4**). The composition of the main peaks was additionally confirmed by isotope distribution analysis (Figures S1 and S2). 

The chemical shift values, δ_P_, of the complexes were −29.4 (**3**) and −27.7 (**4**) ppm. The signals of both complexes in the ^31^P NMR spectra were shifted to the low field by ca. 16 ppm according to the signals of free ligands. The chemical shifts of products and the Δδ values were in the typical range for P,P-chelate copper(I) complexes of 1,5-diaza-3,7-diphosphacyclooctanes [31,33]. 

The ^1^H NMR spectra of the obtained complexes were also very similar. In the spectra of complexes **3** and **4**, four multiplets of pyridyl protons in the ranges 7.47–8.81 (**3**) and 7.48–8.80 ppm (**4**) were registered. The AB systems of phenylene protons were registered at δ 6.81 and 7.11 ppm (**3**), 6.84 and 7.49 ppm (**4**). The methylene protons of the P-CH_2_-N fragments were observed at δ 4.63 and 4.86 ppm (**3**), 4.63 and 4.74 ppm (**4**) in the form of two broad doublets. The methylene protons at C(11) in complex **3** were registered as a singlet at δ 3.62 ppm. The spectra indicated retention of the symmetrical macrocyclic structure of the ligands and similarity of the general structures of these two types of complexes. 

### 2.2. X-ray Diffraction

**Figure 1:**
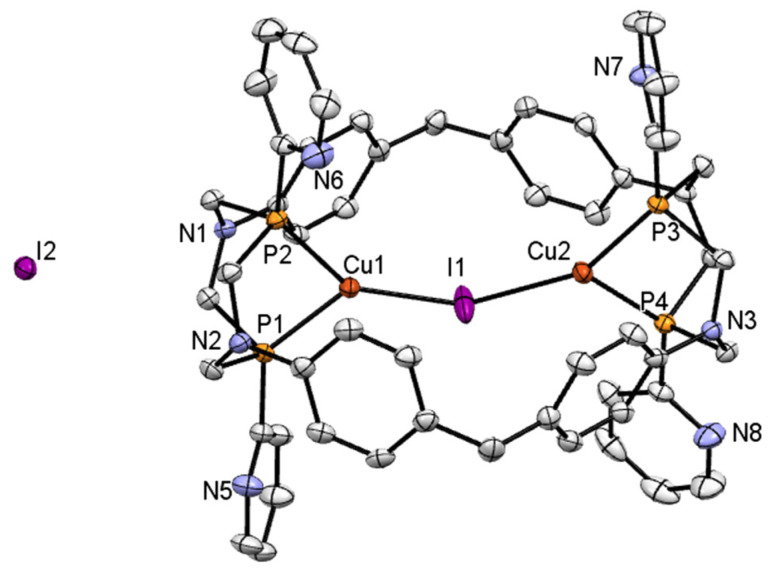
Geometry of complex **3** in a crystal. Solvate DMF molecules and hydrogen atoms are not shown for clarity. Ellipsoids of anisotropic displacements are given with a probability of 50%.

The structure of complex **3** was finally established by X-ray diffraction analysis. The crystals of complex **3** were grown by slow evaporation of the solvent from a solution of complex **3** in DMF. The crystals of complex **3** contained two solvate DMF molecules per one molecule of **3**. This complex appeared to be a cationic binuclear complex where two copper ions were located inside the macrocyclic cavity (Figure 1). 

The metal ions are both trigonal planar (the sums of bond angles are 358.5–360°). Each ion is coordinated by two phosphorus atoms of the 1,5-diaza-3,7-diphosphacyclooctane fragment in the P,P-chelate mode, and these ions are additionally bound by a bridging iodine atom. The bond angle value Cu(1)I(1)Cu(2) (143.83 (3)°) is unusually large as compared with the dimeric bis-P,P-chelate complex of 1,5-bis(diphenylmethyl)-3,7-di(pyridine-2-yl)-1,5-diaza-3,7-diphosphacyclooctane with copper(I) iodide (78.3°) [33], whereas the bond lengths Cu-I are slightly decreased as compared with this complex (2.509 Å vs. 2.64–2.75 Å [33]). There are only two examples of diphosphine complexes with a Cu-I-Cu moiety; in both cases, two copper atoms are situated in close proximity due to the bridging modes of the diphosphine ligands, such as 2,4,6-tris(diphenylphosphino)-1,3,5-triazine [34] or 3,5-di-t-butyl-1,2,4-triphospholyl anion [35]. It should be mentioned that the P-Cu-P angles in both complexes are noticeably smaller than that of **3** (130.5° and 102.3°, correspondingly). The second iodine atom is located in the outer coordination sphere, bonding via hydrogen bonds with protons of endocyclic P-CH_2_-N fragments of two different complex molecules, forming a supramolecular chain structure (Figure S3). 

The conformation of the macrocyclic ligand in complex **3** is only slightly distorted as compared with typical cylindrical conformations of uncoordinated 1,5(1,5)-di(1,5-diaza-3,7-diphosphacyclooctana)-2,4,6,8(1,4)-tetrabenzenacyclooctaphanes [22,32]. The chelating 1,5-diaza-3,7-diphosphacyclooctane fragments maintain the starting chair–chair conformation in contrast to the previously described metal complexes of similar P,N-containing cyclophanes with longer tri- and tetraphenylene phane fragments where the chelating diphosphine rings adopt a chair–boat conformation [26,27].

## 3. Materials and Methods

### 3.1. General

All work with phosphines **1** and **2** was performed in a dry argon atmosphere using the standard Schlenk vacuum technique. The manipulations with complexes **3** and **4** did not require an inert atmosphere. Solvents were purchased from Acros Organics (Geel, Belgium). The solvents used were dried and purified by distillation under inert atmosphere. The ESI_pos_ mass spectra were recorded with an AmazonX (Bruker Daltonics GmbH, Bremen, Germany) spectrometer at a capillary voltage of 4500 V. The mass spectrometry data were solved using the DataAnalysis 4.0 (Bruker Daltonics GmbH, Bremen, Germany) program. The mass spectra are presented as *m/z* values. The ^1^H NMR (400 MHz and 600 MHz) and ^31^P NMR (162 and 242 MHz) spectra were recorded using Bruker Avance-DRX 400 and Bruker Avance-600 spectrometers (BrukerBioSpin, Billerica, MA, USA). The chemical shifts (δ) and coupling constants (*J*) are reported in ppm and in Hz, respectively. The internal standard for ^1^H NMR is SiMe_4_, and the external standard for ^31^P NMR is 85% H_3_PO_4_ (aq). A CHN analyzer “CHN-3 KBA” was used for the determination of the CHN content. The determination of the phosphorus content was provided by combustion in an oxygen stream. The starting pyridylphosphines were prepared according to procedures in the literature.

### 3.2. X-ray Crystallography Data

The X-ray diffraction data for a single crystal of **3** were collected on a single crystal diffractometer Rigaku (Xcalibur 3, Sapphire3, Gemini), using standard procedures (graphite monochromated MoKα (λ = 0.71073 Å) radiation, at temperature 130(2) K, ω-scans with step 1°). A suitable crystal of appropriate dimensions was mounted on glass fibers in a random orientation. For the data collection: Images were indexed and integrated using the CrysAlisPro 1.171.38.43 (Rigaku OD, 2017) programs [36]. Final cell constants were determined by global refinement of reflections from the complete dataset. The structure solution was performed using direct methods with SHELXT-2014/5 [37]. Anisotropic refinement of all non-hydrogen atoms was performed by full matrix least squares on F2 using SHELXL-2017/1 [38]. Calculations were conducted by means of the WinGX-2014.1 suite of programs [39]. All hydrogen atoms were calculated on idealized positions. N atoms of aromatic substituents were located from a bond length analysis. Asymmetric parts of crystal **3** included one molecule of complex and **2** solvate molecules of dimethylformamide. 

The crystal data, data collection, and structure refinement details for the crystal are summarized in Table 1.

Molecular structure of the complex in the crystalline phase as well as accepted partial numbering are presented as ORTEP diagrams from the MERCURY 3.8 program [40]. The crystallographic data for the structure have been deposited in the Cambridge Crystallographic Data Centre as supplementary publication number CCDC 2225171.

### 3.3. Experimental Part

*1^3^,1^7^,5^3^,5^7^-Tetra(pyridine-2-yl)-1,5(1,5)-di(1,5-diaza-3,7-diphosphacyclooctana)-2,4,6,8(1,4)-tetrabenzenacyclooctaphane* (**1**). Bis(4-aminophenyl)methane (0.73 g, 3.69 mmol) in dry DMF (12 mL) was added to a solution of bis(hydroxymethyl)(pyridine-2-yl)phosphine obtained by stirring the mixture of (pyridine-2-yl)phosphine (0.82 g, 7.39 mmol) and paraformaldehyde (0.44 g, 14.67 mmol) at 90 °C up to homogenization, in dry DMF (5 mL). The reaction mixture was stirred at 100–110 °C for 2.5 days and cooled. The precipitate formed was filtered-off, washed with DMF and twice carefully with acetonitrile, and dried for 4 h at 5 × 10^−2^ torr to give **1** as a white powder (0.40 g, 23%). M.p. 224 °C. ^1^H NMR (DMSO-*d*_6_): δ 3.53 (s, 4H, H(11)), 4.32 (dd, ^2^*J*_HH_ = 15.1 Hz, ^2^*J*_PH_ = 5.6 Hz, 8H, H(1)_ax_), 4.53 (dd, ^2^*J*_HH_ = 15.1 Hz, ^2^*J*_PH_ = 9.7 Hz, 8H, H(1)_eq_), 6.66 (d, ^3^*J*_HH_ = 8.7 Hz, 8H, H(8)), 7.09 (d, ^3^*J*_HH_ = 8.7 Hz, 8H, H(9)), 7.32 (ddd, ^3^*J*_HH_ = 7.8 Hz, ^3^*J*_HH_ = 4.8 Hz, ^4^*J*_HH_ = 1.4 Hz, 4H, H(5)), 7.74 (br.d, ^3^*J*_HH_ = 7.6 Hz, 4H, H(3)), 7.78 (ddm, ^3^*J*_HH_ = 7.8 Hz, ^3^*J*_HH_ = 7.6 Hz, ^4^*J*_HH_ = 1.6 Hz, 4H, H(4)), 8.73 (dm, ^3^*J*_HH_ = 4.8 Hz, 4H, H(6)). ^31^P{^1^H} NMR (DMSO-*d*_6_): δ_P_ −45.72 (s). ESI MS: *m/z* 937 [**1** + H]^+^, 959 [**1** + Na]^+^, 982 [**1** + 2Na]^+^, 998 [**1** + Na + K]^+^. Anal. Calc. for C_54_H_52_N_8_P_4_ (936.96): C 69.23, H 5.55, N 11.97, P 13.25%. Found: C 69.78; H 5.81; N 11.61; P 12.80%.

*1^3^,1^7^,5^3^,5^7^-Tetra(pyridine-2-yl)-3,7-dithia-1,5(1,5)-di(1,5-diaza-3,7-diphosphacyclooctana)-2,4,6,8(1,4)-tetrabenzenacyclooctaphane* (**2**). Cyclophane **2** was obtained similar to **1** from (pyridine-2-yl)phosphine (1.04 g, 9.37 mmol), paraformaldehyde (0.56 g, 18.67 mmol), and bis(4-aminophenyl)sulfide (1.01 g, 4.68 mmol). The yield of **2** was 0.35 g (15%). M.p. 222 °C. ^1^H NMR (DMSO-*d*_6_): δ 4.40 (dd, ^2^*J*_HH_ = 15.0 Hz, ^2^*J*_PH_ = 5.7 Hz, 8H, H(1)_ax_), 4.56 (dd, ^2^*J*_HH_ = 15.0 Hz, ^2^*J*_PH_ = 10.5 Hz, 8H, H(1)_eq_), 6.73 (d, ^3^*J*_HH_ = 8.8 Hz, 8H, H(8)), 7.34 (ddd, ^3^*J*_HH_ = 7.6 Hz, ^3^*J*_HH_ = 5.7 Hz, ^4^*J*_HH_ = 1.0 Hz, 4H, H(5)), 7.44 (d, ^3^*J*_HH_ = 8.8 Hz, 8H, H(9)), 7.74 (d, ^3^*J*_HH_ = 7.6 Hz, 4H, H(3)), 7.80 (br.dd, ^3^*J*_HH_ = 7.6 Hz, ^3^*J*_HH_ = 7.6 Hz, 4H, H(4)), 8.74 (br.d, ^3^*J*_HH_ = 4.9 Hz, 4H, H(6)). ^31^P{^1^H} NMR (DMSO-*d*_6_): δ_P_ −43.96 (s). ESI MS: *m/z* 973 [**2** + H]^+^, 995 [**2** + Na]^+^, 1018 [**2** + 2Na]^+^. Anal. Calc. for C_52_H_48_N_8_P_4_S_2_ (973.03): C 64.20, H 4.94, N 11.52, P 12.76, S 6.58%. Found: C 64.67; H 5.17; N 11.38; P 12.30, S 6.28%.

*{[1^3^,1^7^,5^3^,5^7^-Tetra(pyridine-2-yl)-1,5(1,5)-di(1,5-diaza-3,7-diphosphacyclooctana)-2,4,6,8(1,4)-tetrabenzenacyclooctaphane]-μ-iododicopper(I)}iodide* (**3**). Copper(I) iodide (0.0745 g, 0.391 mmol) was added to the solution of **1** (0.1526 g, 0.163 mmol) in dry DMF (25 mL) at 90–100 °C and the reaction mixture was stirred for 42 h at 100 °C. The reaction led to the graduate formation of the yellow fine precipitate which was filtered-off. The mother liquor was concentrated under reduced pressure up to ≈ 1/2 of the initial volume, the precipitate formed was filtered-off, washed twice with acetone, and dried for 3 h at 5 × 10^−2^ torr to obtain complex **3**. The yield of **3** was 0.092 g (43%). ESI MS: *m/z* 1090 [**3**–2I + Na]^+^, 1191 [**3**–I]^+^. Anal. Calc. for C_54_H_52_N_8_P_4_Cu_2_I_2_ (1317.84): C 49.22, H 3.98, N 8.50, P 9.40%. Found: C 48.77; H 3.49; N 8.38; P 9.30%.^1^H NMR (DMSO-*d*_6_): δ 3.58 (s, 4H, H(11)), 4.68 (br.d, ^2^*J*_HH_ = 14.5 Hz, 8H, H(1)_A_), 4.81 (br.d, ^2^*J*_HH_ = 14.5 Hz, 8H, H(1)_B_), 6.73 (d, ^3^*J*_HH_ = 8.8 Hz, 8H, H(8)), 7.13 (d, ^3^*J*_HH_ = 8.8 Hz, 8H, H(9)), 7.50–7.55 (m, 4H, H(5)), 7.64–7.71 (m, 4H, H(3)), 7.90 (dd, ^3^*J*_HH_ ≈ ^3^*J*_HH_ ≈ 8.0 Hz, 4H, H(4)), 8.84 (br.d, ^3^*J*_HH_ = 4.9 Hz, 4H, H(6)). ^31^P{^1^H} NMR (DMSO-*d*_6_): δ_P_ −29.42 (s).

*{1^3^,1^7^,5^3^,5^7^-Tetra(pyridine-2-yl)-3,7-dithia-1,5(1,5)-di(1,5-diaza-3,7-diphosphacyclooctana)-2,4,6,8(1,4)-tetrabenzenacyclooctaphane}bis(copper(I)iodide)* (**4**). Complex **4** was obtained similar to **3** from **2** (0.0510 g, 0.052 mmol) and copper(I) iodide (0.0200 g, 0.104 mmol). The yield of **4** was 0.034 g (48%). ESI MS: *m/z* 1126 [**4**–2I + Na + H]^+^, 1147 [**4**–2I + 2Na + H]^+^, 1191 [**4**–2I + O+(CH_3_)_2_SO]^+^, 1227 [**4**–I]^+^. Anal. Calc. for C_52_H_48_N_8_P_4_S_2_Cu_2_I_2_ (1353.92): C 46.13; H 3.57; N 8.28; P 9.15; S 4.74%. Found: C 46.57; H 3.19; N 8.39; P 9.28, S 4.28%. ^1^H NMR (DMSO-*d*_6_): δ 4.63 (br.d, ^2^*J*_HH_ = 15.2 Hz, 8H, H(1)_A_), 4.74 (br.d, ^2^*J*_HH_ = 15.2 Hz, 8H, H(1)_B_), 6.84 (br.d, ^3^*J*_HH_ = 9.0 Hz, 8H, H(8)), 7.48 (dd, ^3^*J*_HH_ = 5.2 Hz, 4H, H(5), partially overlapped by H(9)), 7.49 (d, ^3^*J*_HH_ = 9.0 Hz, 8H, H(9)), 7.80–7.85 (m, 4H, H(3)), 7.89 (br.dd, ^3^*J*_HH_ ≈ 8.1 Hz, 4H, H(4)), 8.80 (d, ^3^*J*_HH_ = 5.2 Hz, 4H, H(6)). ^31^P{^1^H} NMR (DMSO-*d*_6_): δ_P_ −27.70 (s). 

## 4. Conclusions

The row of 28-membered cyclophanes containing two 1,5-diaza-3,7-diphosphacyclooctane fragments was extended by the synthesis of two new representatives with pyridyl substitutents at the phosphorus atoms. Condensation of pyridine-2-yl-phosphine with paraformaldehyde and (bis(4-aminophenyl)methane or -sulfide resulted in macrocyclic 1,5(1,5)-di(1,5-diaza-3,7-diphosphacyclooctana)-2,4,6,8(1,4)-tetrabenzenacyclooctaphane in lower yields of 15–23% than its P-aryl substitiuted analogues. For the first time, it is shown that the reaction of 28-membered cage cyclophanes containing two 1,5-diaza-3,7-diphosphacyclooctane fragments with two molecules of CuI leads to binuclear P,P-chelate complexes with retention, in general, of the cyclophane geometry. An unusual, charged, and almost linear Cu-I-Cu moiety with trigonal copper atoms is situated within the intramolecular cavity of the cyclophane ligands. 

## Figures and Tables

**Table 1:** Crystal data and structure refinement for complex **3**.

Parameter	3
Moiety formula	C_54_H_52_Cu_2_IN_8_P_4_, 2(C_3_H_7_NO), I
Sum formula	C_60_H_66_Cu_2_I_2_N_10_O_2_P_4_
Formula weight	1464.01
T (K)	130(2)
Crystal system	Monoclinic
Space group	Cc
a (Å)	16.5041(4)
b (Å)	17.3987(4)
c (Å)	21.5078(5)
α (^o^)	90
β (^o^)	104.326(2)
γ (^o^)	90
V (Å^3^)	5983.9(2)
Z	4
d calc (g/cm^3^)	1.625
Absorption coefficient, μ (mm^−1^)	1.902
Max. and min. transmission	1.00000 and 0.67416
Refinement method	Full-matrix least-squares on F^2^
Refleections collected	48,449
Independent reflections	19,153 (R(int) = 0.0539
Data/restraints/parameters	19,153/2/725
Goodness-of-fit	1.0031
Final R indexes [I > 2r (I)]	R_1_ = 0.0472 wR_2_ = 0.1017
Final R indexes [all data]	R_1_ = 0.0583 wR_2_ = 0.1090
Absolute structure parameter	−0.010(8)
Largest diff. peak and hole. (e Å^3^)	2.171 and −1.294 e.Å^−3^

## Data Availability

Not applicable.

## Supplementary Materials

The following supporting information can be downloaded at: https://www.mdpi.com/article/10.3390/molecules28020680/s1, Figure S1: Experimental ESI mass-spectrum for complexes **3**. Theoretical (bottom) isotope distributions of the pick [M − I]^+^ (calc. by https://www.sisweb.com/mstools/isotope.htm (accessed on 2 December 2022)), Figure S2: Experimental ESI mass-spectrum for complex **4**. Theoretical (bottom) isotope distributions of the pick [M − I]^+^ (calc. by https://www.sisweb.com/mstools/isotope.htm (accessed on 2 December 2022)), Figure S3: I…H-C-Contacts forming a supramolecular chain structure of complex **3**.

## Sample Availability

Samples of the compounds **1**–**4** are available from the authors.
