# Supplementary material for: First Example of Cage P4N4-Macrocycle Copper Complexes with Intracavity Location of Unusual Cu2I Fragments
Source: Molecules. 2023 Jan 9;28(2):680. doi: 10.3390/molecules28020680 (PMC9862699; doi:10.3390/molecules28020680)
Supplement: Supplementary file 1 [file molecules-28-00680-s001.zip › molecules-2127230-supplementary.pdf]

molecules

MDPI

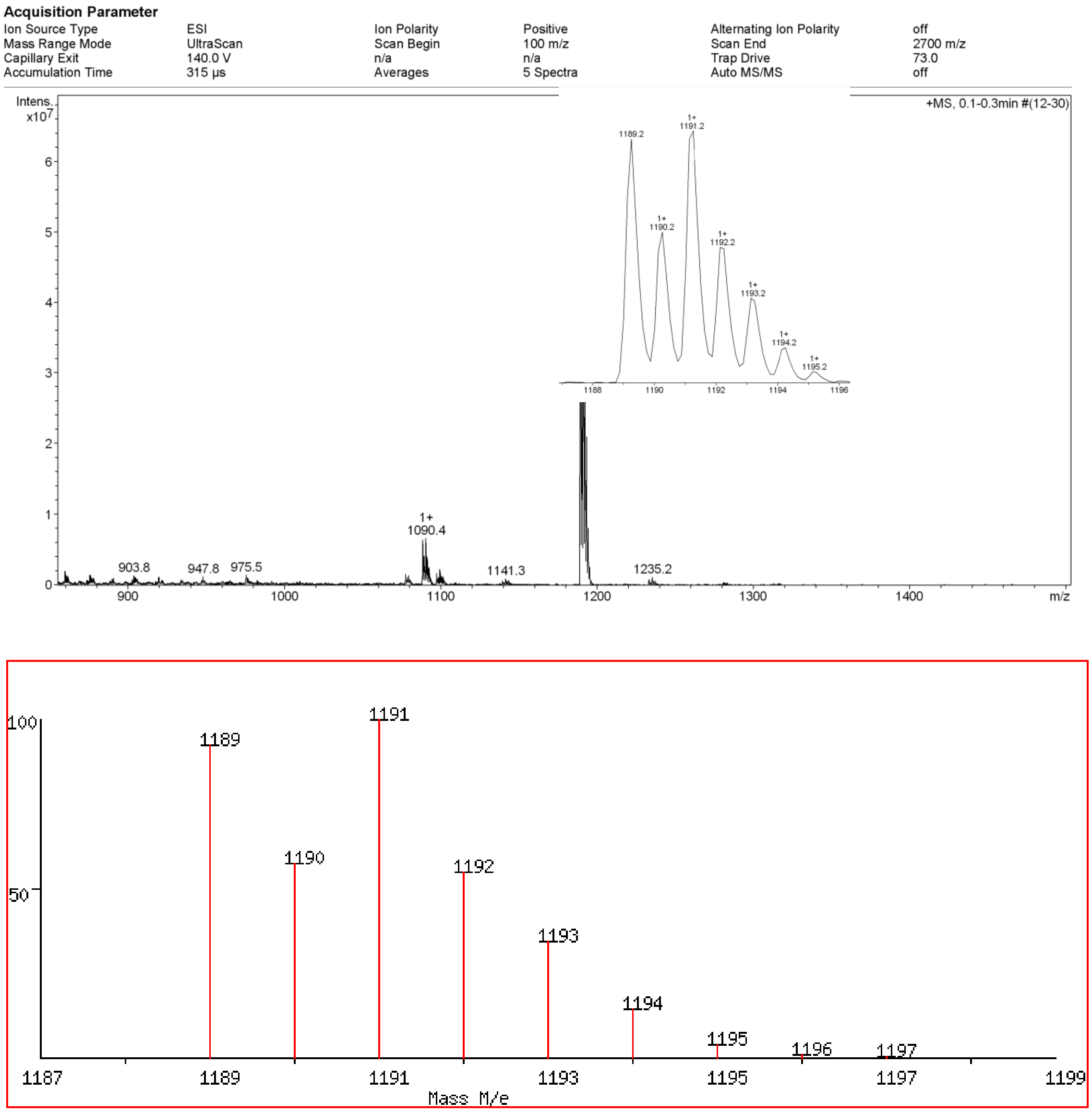


**Figure S1.** Experimental ESI mass-spectrum for complex **3**. Theoretical (bottom) isotope distributions of the pick $[M–I]^+$ (calc. by https://www.sisweb.com/mstools/isotope.htm (accessed on 02 December 2022)).

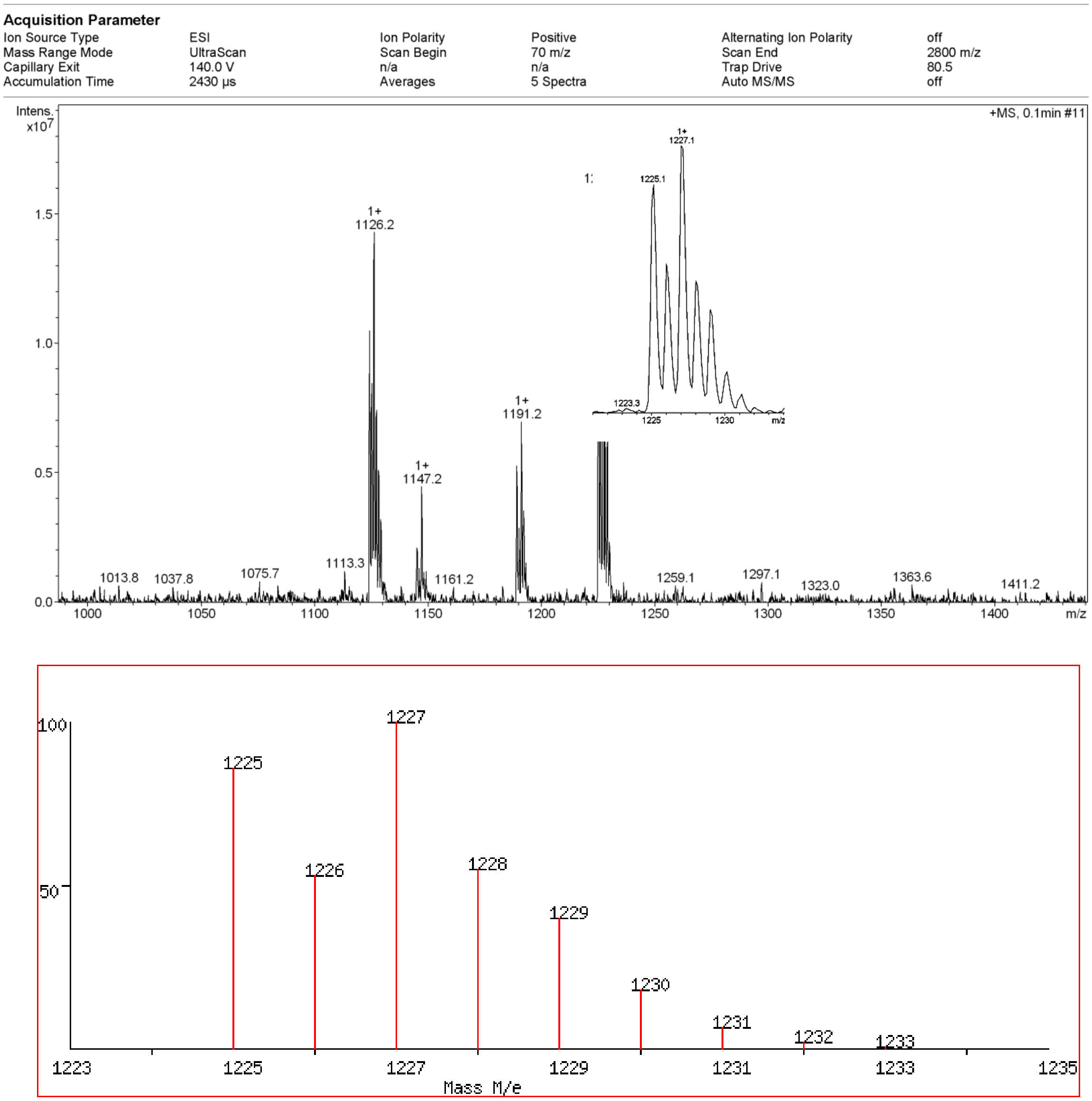


**Figure S2.** Experimental ESI mass-spectrum for complex **4**. Theoretical (bottom) isotope distributions of the pick $[M–I]^+$ (calc. by https://www.sisweb.com/mstools/isotope.htm (accessed on 02 December 2022)).

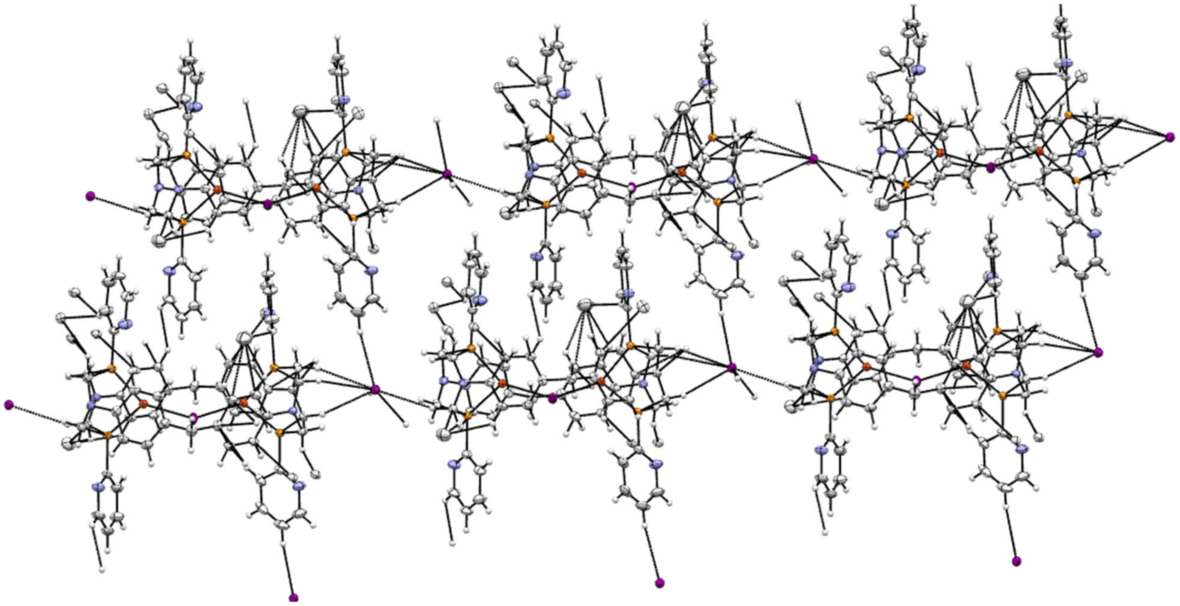

**Figure S3.** I…H-C-Contacts forming a supramolecular chain structure of complex **3**.
